# The significance of consolidation chemotherapy after concurrent chemoradiotherapy in esophageal squamous cell carcinoma: a randomized controlled phase III clinical trial

**DOI:** 10.1111/1759-7714.15424

**Published:** 2024-08-28

**Authors:** Qingshan Zhu, Chi Zhang, Zhuoqi Li, Tingwei Ma, Nengchao Wang, Weipeng Liu, Zhijie He, Jing Shen, Tao Wei, Shijie Zhao, Lianjie Feng, Yuan Tian

**Affiliations:** ^1^ Radiotherapy Department Anyang Cancer Hospital of Henan Province Anyang China; ^2^ Radiotherapy Department, Anyang Cancer Hospital affiliated with Henan University of Science and Technology Anyang China; ^3^ Radiotherapy Department Henan Provincial Key Laboratory of Precision Prevention and Treatment of Esophageal Cancer Anyang China; ^4^ Department of Cardiology The Second Hospital, Cheeloo College of Medicine, Shandong University Jinan China; ^5^ Department of Radiotherapy Oncology Affiliated Hospital of Shandong University of Traditional Chinese Medicine Jinan China

**Keywords:** concurrent chemoradiotherapy, consolidation chemotherapy, esophageal squamous cell carcinoma

## Abstract

**Background:**

This study explored the significance of consolidation maintenance chemotherapy after concurrent chemoradiotherapy with different regimens in patients with esophageal squamous cell carcinoma.

**Method:**

A prospective randomized controlled phase III clinical trial was designed and registered in the China Clinical Trials Registry (Registration number: ChiCTR‐TRC‐12002719). Survival data were analyzed in terms of intention‐to‐treat (ITT) and per‐protocol (PP) sets for patients undergoing cisplatin and 5‐fluorouracil (PF) (group A), or cisplatin and paclitaxel (TP) (group B).

**Results:**

The incidence risk of grade III–IV leukopenia in group B was higher than in group A (49.2% vs. 25.5%, *p* = 0.012). The survival rates at 1, 2, 3, and 5 years were 83.8%, 62.6%, 53.1%, and 41.3%, respectively. Consolidation chemotherapy after concurrent chemoradiation therapy had no benefit on median progression‐free survival (PFS) (*p* = 0.95) and overall survival (OS) (*p* = 0.809). According to the ITT analysis, the median PFS in group A and group B was 28.6 months and 30.3 months (*X* ^2^ = 0.242, *p* = 0.623), while the median OS was 31.0 months and 50.3 months (*X* ^2^ = 1.25，*p* = 0.263). For the PP analysis, the median PFS in group A and group B were 28.6 months and 30.3 months (*p* = 0.584), while the median OS was 31.0 months and 50.3 months (*p* = 0.259), respectively. Patients receiving consolidation chemotherapy did not show significant OS benefits (46.9 months vs. 38.3 months; *X* ^2^ = 0.059, *p* = 0.866).

**Conclusion:**

Similar PFS and OS were found between PF and TP regimens with concurrent chemoradiotherapy. Consolidation chemotherapy did not show any significant OS benefits.

## Graphical abstract

To explore the significance of consolidation chemotherapy in patients with ESCC, a prospective randomized controlled phase III clinical trial was designed. Survival data was analyzed for patients undergoing PF, or TP regimen. It was found that consolidation chemotherapy did not show any significant OS benefits.

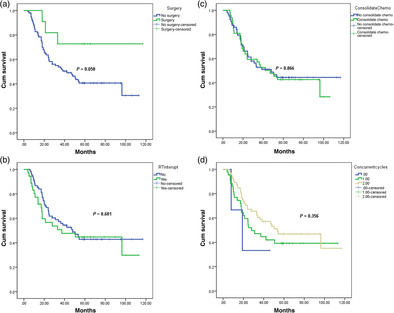

## INTRODUCTION

Worldwide, the number of patients with esophageal squamous cell carcinoma (ESCC) ranks seventh in terms of cancer incidence and sixth in terms of mortality, with significant variations in tumor pathology and morphology, and geographical distributions.^1^ China is a high‐incidence area of ESCC, and although its incidence and mortality have slightly decreased in recent years, the death‐to‐incidence ratio has remained between 0.74 and 0.76.^2^, ^3^, ^4^, ^5^ Moreover, there are also notable regional differences in incidence, particularly in the Taihang Mountain District, which is located at the junction of Henan, Hebei, and Shanxi and is considered a high‐incidence area worldwide.^6^, ^7^ Additionally, ESCC is known for its poor prognosis in cases of late diagnosis and due to a lack of effective treatment.^1^, ^8^

Concurrent chemoradiotherapy is the standard of care for patients who are unable or unwilling to undergo surgery, and the fluorouracil plus cisplatin (PF) regimen is considered the first‐line standard chemotherapy.^9^, ^10^ In recent years, the combination of paclitaxel and cisplatin (TP) with radiotherapy has been increasingly used in clinical settings, and it has been shown to have higher short‐term efficacy compared to traditional treatment regimens.^11^, ^12^ This study aimed to compare the prognostic differences of PF and TP regimens in the treatment of ESCC in combination with concurrent radiotherapy. The objective was to determine the more suitable chemoradiotherapy regimen for patients with ESCC. The study was designed and registered in the China Clinical Trials Registry (Registration number: ChiCTR‐TRC‐12002719).

## METHODS

### Data collection

This study was a single‐center, prospective, randomized controlled clinical trial, registered in the China Clinical Trials Registry on 27 November, 2012 (ChiCTR‐TRC‐12002719). The protocol was approved by the Ethics Committee of Anyang Cancer Hospital in Henan Province, and all patients signed informed consent.

#### Inclusion criteria

The inclusion criteria in this study were: (1) patients aged 18–70 years with a pathological diagnosis of ESCC; (2) locally advanced and inoperable disease or deemed unsuitable for surgery due to other diseases or unwillingness to undergo surgery; (3) Eastern Cooperative Oncology Group status of 0–1; (4) previous chemotherapy recipients requiring a 4‐week washout period; and (5) clinical staging determined according to the draft clinical staging criteria for nonsurgical treatment of ESCC.^13^

#### Baseline assessments

Mandatory assessments included complete blood count, blood biochemistry, electrocardiogram, esophageal angiography, gastroscopic pathological biopsy, and chest and upper abdomen computed tomography (CT). Magnetic resonance imaging, emission computed tomography, and positron emission tomography‐computed tomography were not mandatory tests.

#### Grouping method

The random numbers were generated using a random number generator, with odd numbers assigned to group A and even numbers assigned to group B.

#### Endpoints

The primary endpoint was progression‐free survival (PFS), while the secondary endpoint was overall survival (OS).

### Treatment regimens

#### Chemotherapy regimens

Group A: Cisplatin 20 mg/m^2^, intravenous (IV), day1‐5; 5‐fluorouracil 500 mg/m^2^, IV, 4–6 h, d1‐5. Group B: Cisplatin 20 mg/m^2^, IV, day1‐5; paclitaxel 80 mg/m^2^, IV, day1‐8, with routine antiallergic pretreatment. Both groups received two cycles of chemotherapy during the first and fifth weeks of radiotherapy. A time error of up to 3 days was allowed during implementation.

#### Radiotherapy regimens

The radiotherapy regimen was the same in both groups.^14^

##### Target area determination

The target area was determined by chest CT and esophageal angiography, with the length of esophageal irradiation extending 3 cm beyond the upper and lower boundaries of the tumor.

##### Radiotherapy planning

Based on CT simulation positioning and a three‐dimensional planning system, intensity‐modulated radiation therapy (IMRT) was utilized.

##### Radiotherapy dose

DT50.4Gy/28f, 1.8Gy/f, 1f/day, 5 days/week, for a total of 6 weeks.^14^

##### Organ protection and dose restriction

Whole lung: V20 ≤ 30%, V30 ≤ 20%, V5 ≤ 60%, MLD <13Gy. Spinal cord: *D*_max_ ≤45Gy. Heart: V40 ≤ 40%, D100 ≤ 30Gy, D50 < 40Gy. Liver: D60 < 30Gy, *D*_mean_ <25Gy.

#### Dose adjustment

The dose of drugs and radiotherapy was adjusted within a 25% dose range based on the severity of adverse reactions. If a patient developed intolerable adverse effects, chemotherapy was discontinued while radiation therapy was given alone. However, subsequent consolidation chemotherapy was not restricted.

#### Adjunctive supportive care

Routine preventive medication and anti‐allergy pretreatment of gastrointestinal reactions were implemented. There were no restrictions on traditional Chinese medicines, except for immunological preparations and antitumor drugs.

#### Evaluation of tumor treatment efficacy

According to the Response Evaluation Criteria In Solid Tumors (RECIST) trial 1.1, the tumor treatment efficacy, including complete response (CR), partial response (PR), stable disease (SD), and progressive disease (PD), were evaluated.

#### Security assessments and analyses

Adverse reactions were assessed according to the Common Terminology Criteria for Adverse Events (CTCAE 3.1). Additionally, safety was assessed and analyzed in all enrolled and treated patients.

#### Patient follow‐up

A combination of hospital visits and telephone follow‐up was used, with follow‐up performed once every 3 months. At least one successful follow‐up visit was completed for inclusion in the final comprehensive analysis.

PFS was defined as the time from randomization to disease progression at any site and OS was defined as the time from randomization to death from any cause or cut‐off in the follow‐up. PFS and OS were analyzed using intention‐to‐treat (ITT) populations and per‐protocol (PP) populations. In accordance with clinical practice, the following criteria were considered to be in line with the protocol: (1) radiotherapy dose ≥50Gy; (2) completion of at least one cycle of chemotherapy.

#### Statistical analysis

Statistical Package for the Social Sciences version 20.0 software (IBM Corp.) was used for the statistical analysis of the study data. Furthermore, Kaplan–Meier survival analysis was carried out to compare PFS and OS between the groups using a log‐rank test. The comparison of counting data was calculated by the *X* ^2^ test. The comparison of categorical data was assessed using the chi‐square test, while the comparison of the ordinal data was performed by the Wilcoxon rank sum test. The two‐sided test was used, with a significant test level at *α* = 0.05.

## RESULTS

### Enrollment results

**FIGURE 1:**
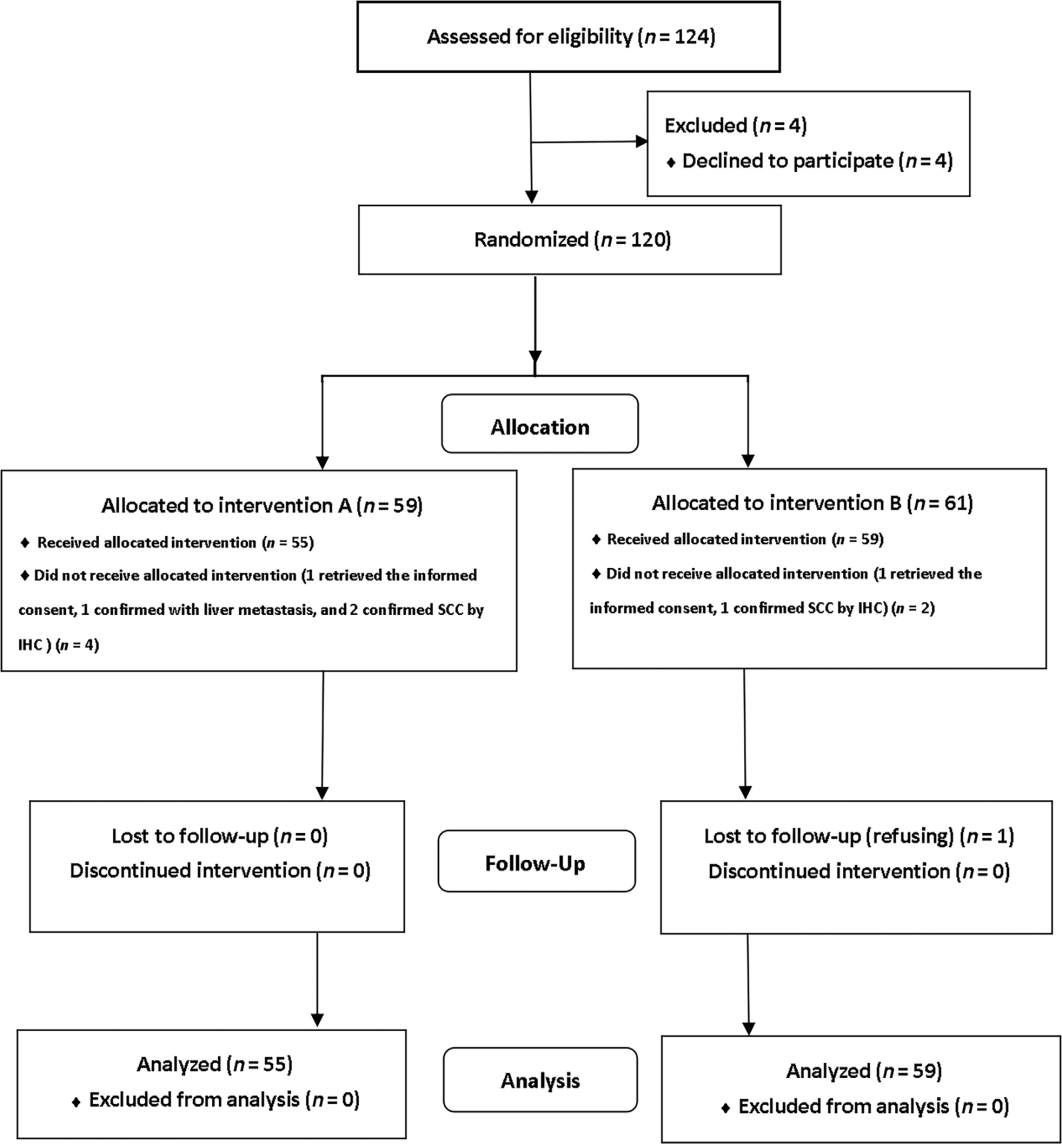
The flow diagram of the study.

From December 2012 to November 2018, a total of 124 patients were screened and 120 were randomized for grouping. After grouping, two cases in group A were excluded due to pathological consultation indicating small cell carcinoma, one case had liver metastases, and one case was excluded from the group due to withdrawal of informed consent. One case in group B was excluded from the group after pathological consultation revealed small cell carcinoma. Finally, 114 patients were enrolled and evaluated for adverse reactions, with 55 in group A and 59 in group B. The consort flow diagram is provided in Figure 1.

### Clinical characteristics

The median age in group A was 64 years, while it was 62 years in group B. No significant differences were found in terms of gender, age, lesion location, clinical stage, or completion of treatment regimens between the two groups (*p* > 0.05) (Table 1). Five patients in each group underwent surgery, all of which were performed after consultation by a multidisciplinary team. Twenty‐five patients in each group received consolidation chemotherapy after completing concurrent chemoradiotherapy. A total of 46 patients received reduced chemotherapy. Thirteen patients received radiation doses below 50 Gy, of which 10 were for surgical treatment.

**TABLE 1:** Basic characteristics of all enrolled patients.

Variable	No. (%)		*p* value
	Group A (*n* = 55)	Group B (*n* = 59)	
Age (years), Median	64	62	0.100
<70 years	53 (96.4)	57 (96.6)	
≥70 years	2 (3.6)	2 (3.4)	
Sex			0.148
Male	37 (67.3)	47 (79.7)	
Female	18 (32.7)	12 (20.3)	
Blood type			0.118
A	15 (27.3)	10 (16.9)	
B	18 (32.7)	20 (33.9)	
O	15 (27.3)	14 (23.7)	
AB	1 (1.8)	7 (11.9)	
Unknown	6 (10.9)	8 (13.6)	
Alcohol	17 (30.9)	15 (25.4)	0.422
Smoker	27 (49.1)	35 (59.3)	0.312
Family history (ESCC)	16 (29.1)	16 (27.1)	0.702
ECOG status			0.632
0	39 (70.9)	37 (62.7)	
1	16 (29.1)	22 (37.3)	
BMI			0.939
Median	22.8 (21.9–23.9)	23.5 (22.4–23.9)	
<18.5	1 (1.8)	3 (5.1)	
≥18.5	54 (98.2)	56 (94.9)	
NRS‐2002			0.642
<3	53 (96.4)	57 (96.6)	
≥3	2 (3.6)	2 (3.4)	
Charlson Comorbidity Index			0.285
0–1	12 (21.8)	19 (32.2)	
≥2	43 (78.2)	40 (67.8)	
Tumor length (cm)			0.663
≤5	29 (52.7)	27 (45.8)	
>5	26 (47.3)	32 (54.2)	
Tumor maximum diameter (cm)			0.465
≤2	14 (25.5)	13 (22.0)	
>2	41 (74.5)	46 (78.0)	
Tumor location			0.919
Upper thoracic	34 (61.8)	37 (62.7)	
Middle thoracic	20 (36.4)	22 (37.3)	
Lower thoracic	1 (1.8)	0 (0)	
HB (g/L) (mean)	134.1 ± 16.1	135.1 ± 17.6	0.878
≥10	54	59	
<10	1	1	
WBC (×10^9^/L) (mean)	6.4 ± 1.5	6.6 ± 1.8	0.536
PLT (×10^9^/L) (mean)	231.2 ± 49.6	226.1 ± 63.7	0.636
RBC (×10^12^/L) (mean)	4.3 ± 0.5	4.3 ± 0.5	0,845
Stage (AJCC, 6th edition)			0.901
I	6 (10.9)	8 (13.5)	
II	24 (43.6)	25 (42.4)	
III	24 (43.6)	24 (40.7)	
IVA	1 (1.8)	2 (3.4)	
Per protocol	49 (89.1)	53 (89.8)	0.107
Surgery	5 (9.1)	5 (8.5)	0.930
Induce chemotherapy	5 (9.1)	5 (8.5)	0.906
Consolidate chemotherapy	25 (45.5)	25 (42.4)	0.865

Abbreviations: AJCC, American Joint Committee on Cancer; BMI, body mass index; ECOG, Eastern Cooperative Oncology Group; ESCC, esophageal squamous cell carcinoma; HB, hemoglobin; NRS‐2000, nutrition risk screening‐2000; PLT, platelet; RBC, red blood cell.

### Therapeutic efficacy evaluation

The short‐term efficacy of CR, PR, SD, and PD in the two groups were 1, 50, 4, 0 in group A, and 8, 50, 1, 0 in group B, respectively. The CR rates were 1.8% (group A) compared to 13.6% (group B). The PR rates were 90.9% (group A) compared to 84.7% (group B). The response rates (CR + PR) were 92.7% (group A) compared to 98.3% (group B). No cases of PD were found, and the disease control rate (CR + PR + SD) was 100% in both groups (Supporting Information Figure S1).

### Adverse events evaluation

There were no treatment‐related deaths among the 114 patients. A total of five patients developed esophageal fistula, with two cases occurring during radiotherapy and three cases occurring within 1 month after the end of treatment. For the two patients who developed esophageal fistula, one patient completed the radiotherapy plan after the fistula self‐repaired on stopping radiotherapy (Supporting Information Figure S2a,b), while the other patient underwent nasal placement of a duodenal nutrition tube for supportive treatment, with a PFS of 10 months (Supporting Information Figure S2c,d). The incidence of grade III–IV leukopenia in group B was higher than in group A (49.2% [29/59] vs. 25.5% [14/55]), and the difference was statistically significant (*X* ^2^ = 6.805, *p* = 0.012). However, there was no statistically significant difference in other adverse reactions between the two groups (*p* > 0.05) (Table 2).

**TABLE 2:** Treatment‐related Adverse Events (Safety Population).

Event	Group A (*N* = 55)		Group B (*N* = 59)	
	Any grade (*n*, %)	Grade 3 or 4 (*n*, %)	Any grade (*n*, %)	Grade 3 or 4 (*n*, %)
Leukopenia	51 (92.7)	14 (25.5)	56 (94.9)	29 (49.2)
Decreased appetite	50 (90.9)	8 (14.5)	49 (83.1)	6 (10.2)
Esophagitis	50 (90.9)	7 (12.7)	53 (89.8)	9 (15.3)
Alopecia	49 (89.1)	0 (0)	59 (100)	0 (0)
Fatigue	40 (72.7)	3 (5.5)	46 (79.0)	3 (5.1)
Constipation	38 (69.1)	2 (3.6)	42 (71.2)	5 (8.5)
Radiodermatitis	37 (67.3)	1 (1.8)	43 (72.9)	1 (1.7)
Nausea	36 (65.5)	5 (9.1)	41 (69.5)	5 (8.5)
Vomiting	21 (38.2)	2 (3.6)	26 (44.1)	0 (0)
Anemia	11 (20.0)	1 (1.8)	21 (35.6)	2 (3.4)
Thrombocytopenia	8 (14.5)	1 (1.9)	8 (13.6)	2 (3.4)
Fistula	4 (7.3)	0 (0)	1 (1.7)	0 (0)
Diarrhea	3 (5.5)	0 (0)	4 (6.8)	0 ()
Pneumonitis	3 (5.5)	0 (0)	3 (5.1)	0 (0)
Febrile neutropenia	1 (1.8)	0 (0)	3 (5.1)	0 (0)
Infection	0 (0)	0 (0)	0 (0)	0 (0)

### Survival analyses results

#### Survival of the whole group

**FIGURE 2:**
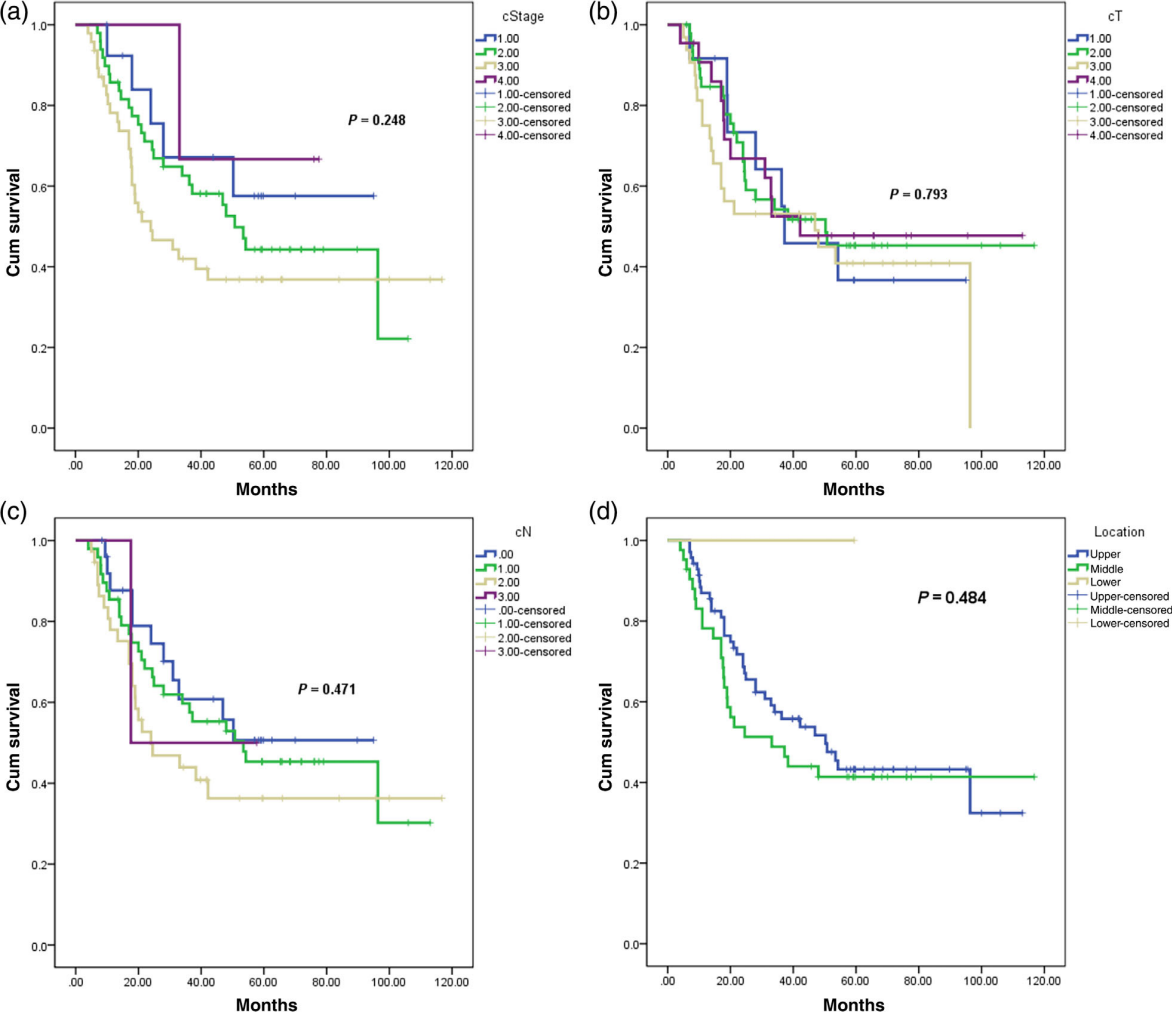
The comprehensive survival analysis results of all enrolled patients. The horizontal axis represents the survival time; The vertical axis represents the survival probability. A: The results of survival analysis based on different clinical stages; Different colored curves represent different clinical stages. B: Results of survival analysis based on different T‐stages; Different colored curves represent different T‐stages. C: Results of survival analysis based on different N‐stages; Different colored curves represent different N‐stages. D: Results of survival analysis based on different locations; Different colored curves represent different locations.

As of August 31, 2022, a total of 113 cases were selected for survival analysis (one case was lost in group B), as displayed in Figure 2. The median follow‐up period was 62.58 months, while the median survival was 46.9 months (95% confidence interval [CI] 28.956–64.844). The mean survival time was (60.43 ± 4.72) months. The estimated survival rates for 1, 2, 3, and 5 years were 83.8%, 62.6%, 53.1%, and 41.3%, respectively.

#### Survival analyses by ITT

**FIGURE 3:**
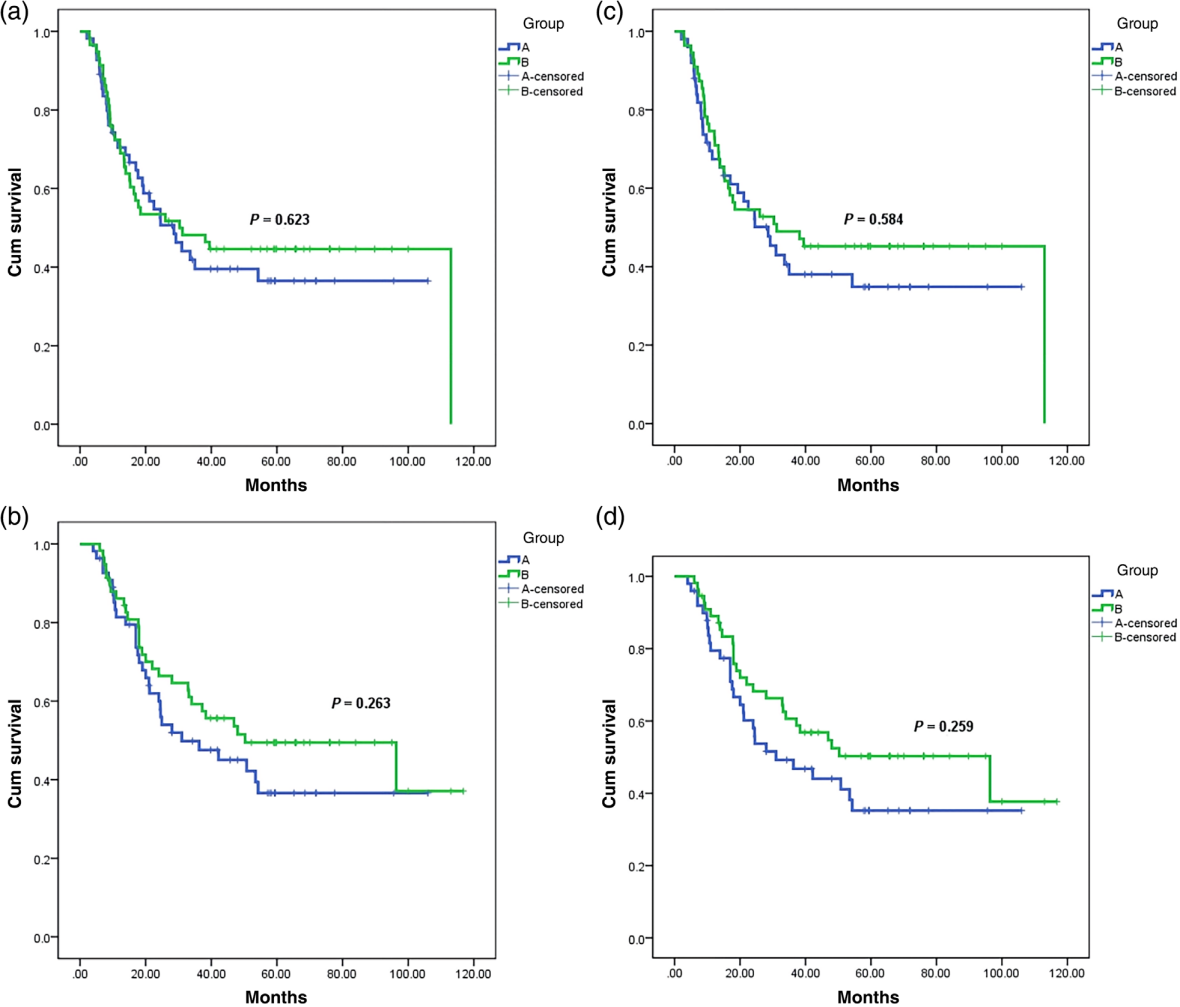
The comprehensive survival analysis results of groups A and B patients. The horizontal axis represents the survival time; The vertical axis represents the survival probability; The blue curve represents group A; The green curve represents group B. A: Results of PFS survival analysis based on ITT. B: Results of overall survival analysis based on ITT. C: Results of PFS survival analysis based on PP. D: Results of overall survival analysis based on PP.

The median PFS was 28.6 months (95% CI 17.99–39.21) in group A and 30.33 months (95% CI 4.00–56.66) in group B, with no significant difference found between the two groups (*X* ^2^ = 0.242, *p* = 0.623) (Figure 3a). The median OS was 31.0 months (95% CI 4.79–51.21) in group A and 50.27 months (95% CI 14.75–85.79) in group B, with no significant difference found between the two groups (*X* ^2^ = 1.25, *p* = 0.263) (Figure 3b).

#### Survival analyses by PP

One hundred and four patients followed the protocol, including 50 in group A and 54 in group B. The median PFS was 28.6 months (95% CI 18.33–38.87) in group A and 30.33 months (95% CI 4.96–55.70) in group B, with no significant difference found between the two groups (X^2^ = 0.299, *p* = 0.584) (Figure 3c). The median OS was 31.0 months (95% CI 9.51–52.49) in group A and 50.27 months (95% CI 14.71–85.84) in group B, with no significant difference found between the two groups (*X* ^2^ = 1.276, *p* = 0.259) (Figure 3d).

#### The impact of surgery, consolidation chemotherapy, radiotherapy interruption, and concurrent chemotherapy on survival

**FIGURE 4:**
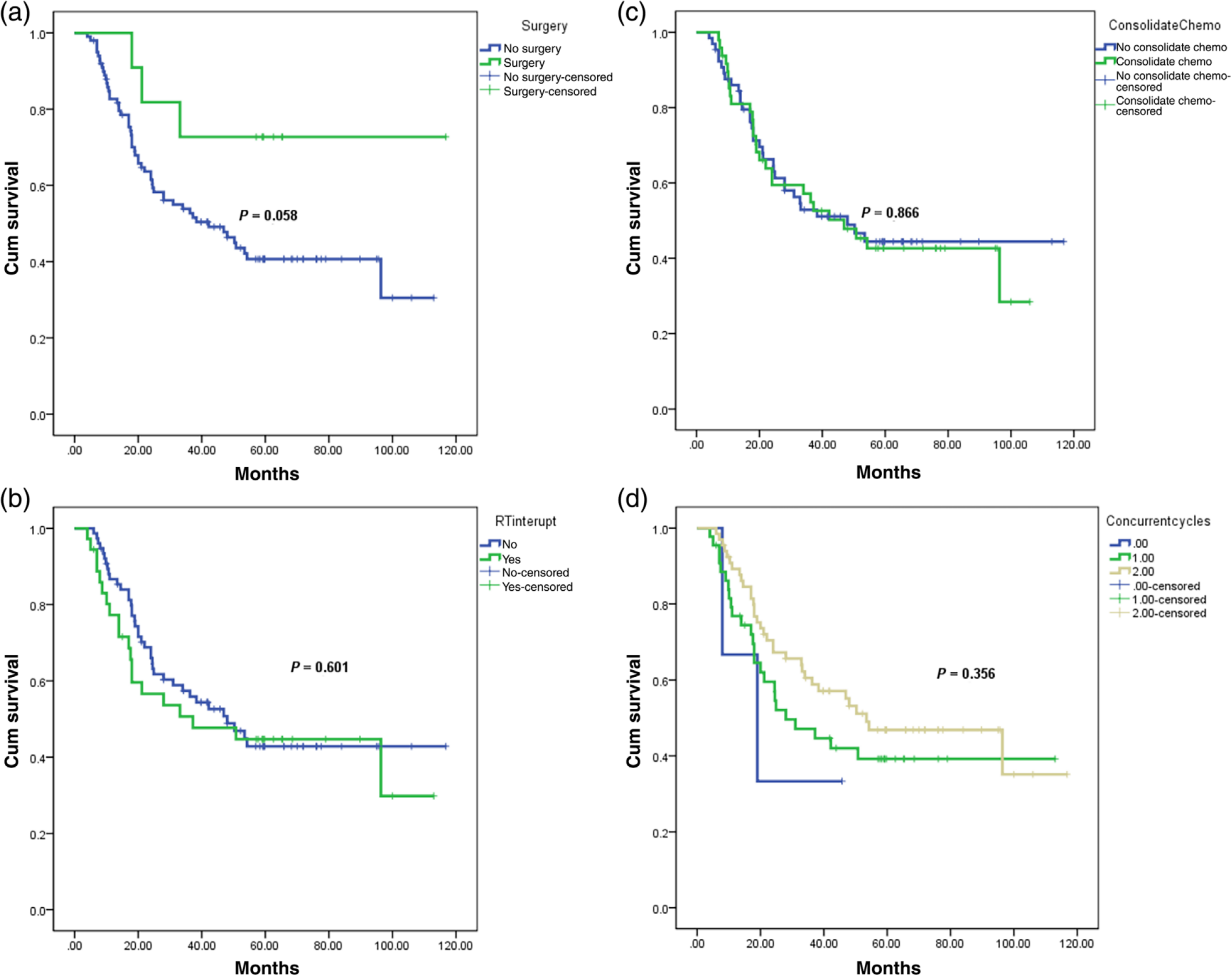
The results of comprehensive survival analysis based on relevant prognostic risk factors. The horizontal axis represents the survival time; The vertical axis represents the survival probability. A: The results of survival analysis based on surgery or not; The green curve represents receiving surgical treatment; The blue curve represents receiving no surgical treatment. B: The results of survival analysis based on RT interruption or not; The green curve represents the occurrence of RT interruption; The blue curve represents that no RT interruption has occurred. C: The results of survival analysis based on consolidation or not; The green curve represents receiving consolidation chemotherapy; The blue curve represents receiving no consolidation chemotherapy. D: The results of survival analysis based on concurrent cycles. Different colors represent different concurrent cycles.

Eleven patients underwent surgery, eight of whom were patients who required surgery after half the volume dose of radiotherapy and three were salvage surgery after relapse. The median survival time had not yet been reached. The median OS time for patients who did not undergo surgery was 37.23 months (95% CI 18.15–56.31) (*X* ^2^ = 3.584, *p* = 0.058) (Figure 4a).

The median OS time was 37.23 months (95% CI 0.0, 78.86) for patients with interrupted radiotherapy (not completing radiotherapy as planned) and 48.0 months (95% CI 29.38, 66.62) for patients without interrupted radiotherapy, with no statistically significant difference found (*X* ^2^ = 0.273, *p* = 0.601) (Figure 4b).

The median OS time of patients receiving consolidation chemotherapy was 46.9 months (95% CI 26.60, 67.20), while the median OS time of patients not receiving consolidation chemotherapy was 38.32 months (95% CI 13.50, 63.14), with no statistically significant difference found (*X* ^2^ = 0.059, *p* = 0.866) (Figure 4c).

During the treatment process, patients who did not undergo concurrent chemotherapy as planned had a median OS time of 19.0 months (95% CI 1.28, 36.72), patients who received one cycle of concurrent chemotherapy had a median OS time of 28.0 months (95% CI 12.24, 43.76), and patients who completed two cycles of concurrent chemotherapy as planned had a median OS time of 53.46 months (95% CI 21.60, 85.32), with no statistically significant difference found (*X* ^2^ = 2.063, *p* = 0.356) (Figure 4d).

#### The impact of multivariate analysis on survival

**FIGURE 5:**
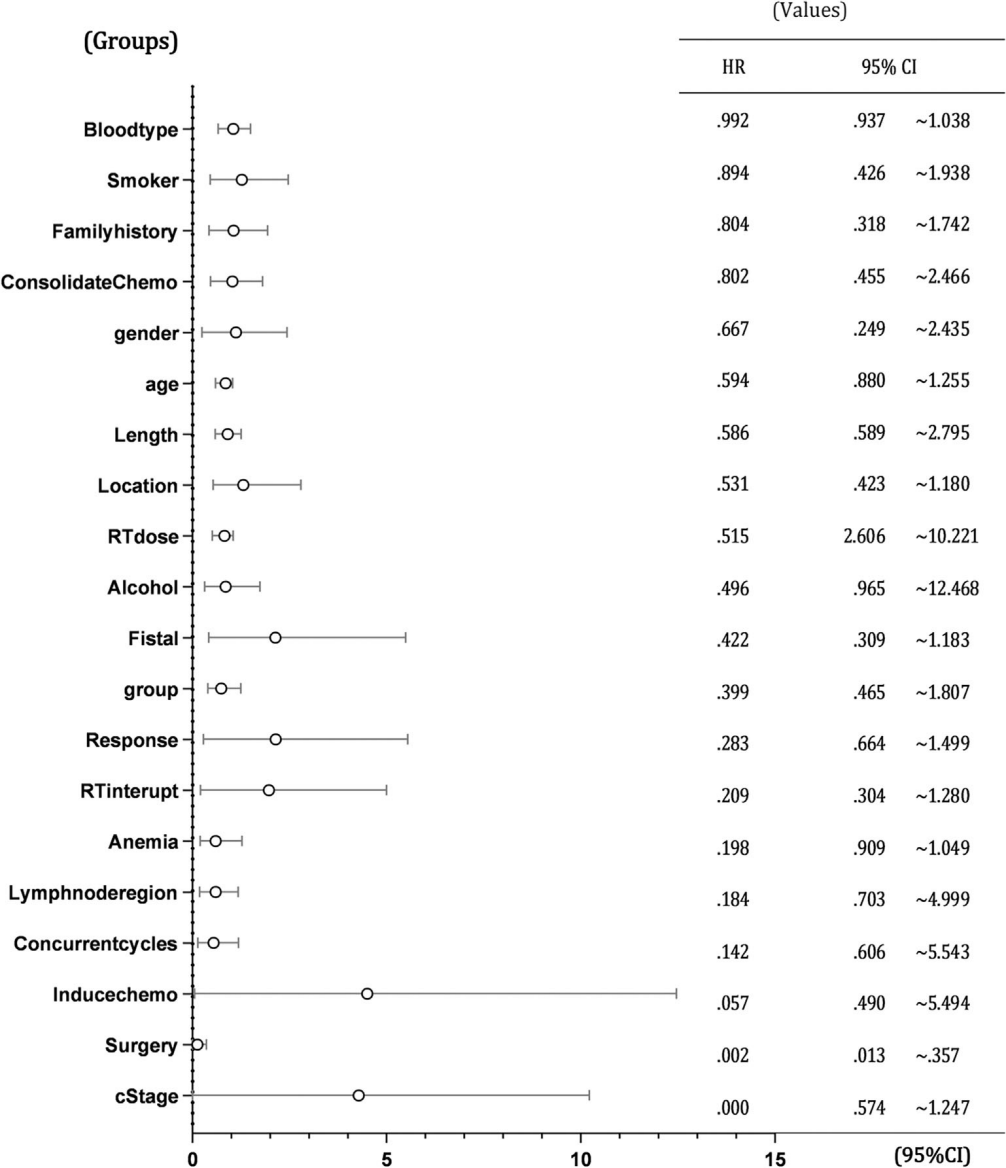
The impact of multivariate analysis on survival. The vertical axis in the left column represents different risk factors; The right column represents hazard ratio (HR) and 95% confidence interval (CI).

Multivariate regression analysis indicated no correlation between gender, age, different chemotherapy regimens, family history, smoking and alcohol history, location, length and diameter of tumor lesions, lymph node involved area, induced chemotherapy, number of concurrent chemotherapy cycles, consolidation chemotherapy, ABO blood group, pre‐treatment hemoglobin levels, and survival time. Clinical stage was identified as a prognostic factor, and patients who received surgical treatment had a significant survival advantage compared to those who did not receive surgery (Figure 5).

## DISCUSSION

At present, the treatment regimen for esophageal cancer is mostly recommended by the guidelines of the National Comprehensive Cancer Network. However, the proportion of adenocarcinoma is higher in Western countries, and it remains to be further studied whether the recommended radiotherapy dose and chemotherapy regimen are suitable for Chinese patients. The 5‐year survival rate of esophageal cancer has been increasing year by year, partly due to the improvement in early diagnosis.^15^ Concurrent chemoradiotherapy as the standard therapy regimen for patients with unresectable esophageal cancer was established by the RTOG85‐01 trial.^16^ However, there are large differences in the sensitivity of concurrent chemoradiotherapy in the real world.^17^ Despite the improvement in the survival rate with IMRT, overall efficacy has remained unsatisfactory, with a 5‐year survival rate of around 20%.^13^, ^15^ The PF regimen has been considered the first‐line standard chemotherapy regimen in concurrent chemoradiotherapy; Paclitaxel‐based combination chemotherapy regimens have been reported to provide a better survival advantage but with a higher incidence risk of grade III to IV adverse reactions.^18^ However, our study found no differences in short‐term efficacy were found between the two treatment regimens, therefore further evaluation of the PFS and OS was necessary, and this study was designed and put into practice (Figure 1).

Baseline characteristics of all enrolled patients are provided in Table 1 and no intergroup differences affecting the analysis results were found (*p* ≥ 0.1). Compared to PF, this study indicated that TP increased severe myelosuppression (Table 2), which is consistent with previous reports.^18^, ^19^ However, there was no statistically significant difference in PFS and OS between the two protocols (Figure 3), according to either ITT or PP.^18^, ^19^ The clinical guidance significance of this study is to facilitate individualized treatment options; For example, in patients with clinical allergies, diabetes mellitus, or impaired glucose tolerance, paclitaxel‐free regimens were preferred to avoid excessive glucocorticoid use. Moreover, PF was a safer option with fewer toxic side effects (Table 2).^18^ The overall 5‐year survival rate for the whole group reached 41.3%, and we speculated that the benefit was related to the use of concurrent chemoradiotherapy,^16^, ^17^ as well as the significantly longer survival of patients who underwent surgery after chemoradiotherapy.^20^, ^21^, ^22^

Esophageal fistula is still a side effect of radiotherapy that we need to be vigilant about during the treatment process (Supporting Information Figure S2), especially for patients with locally advanced ESCC.^23^, ^24^, ^25^ In this study, five patients suffered from esophageal fistula (Supporting Information Figure S2), two of which occurred during treatment and three occurred within 1 month of treatment completion. On further review of the clinical data of the five patients, we found that the tumor length of two patients was greater than 10 cm, suggesting that the length of esophageal lesions might also be a high‐risk factor for esophageal fistula in patients receiving radiotherapy. This finding emphasizes the need for vigilance regarding the risk of esophageal fistula in patients with longer esophageal lesions undergoing radiotherapy.

Consolidation therapy or maintenance therapy is a common treatment method for advanced cancer patients, especially with the emergence of immunotherapy drugs.^26^, ^27^, ^28^, ^29^ However, compared with neoadjuvant chemoradiotherapy,^20^, ^22^, ^30^ there are few reports of consolidation chemotherapy for locally advanced esophageal cancer. Disappointingly, neither chemotherapy regimen showed a statistically significant impact on the survival of patients with consolidation chemotherapy (Figure 4c). Meanwhile, the momentary interruption of radiotherapy did not show any significant impact on the patient's survival time (Figure 4b). This suggested that when the patient's general condition was poor, radiotherapy could be temporarily interrupted, which would not affect the patient's survival time. Patients who completed concurrent chemoradiotherapy showed a trend of survival benefits, which was consistent with previous reports.^20^, ^22^, ^30^

Multivariate regression analysis revealed that clinical stage remained an important prognostic factor, and patients who received surgical treatment had a significant survival advantage compared to those who did not undergo surgery (Figure 5). These findings are consistent with the conclusions drawn from previous clinical studies,^20^, ^21^, ^22^, ^30^ which further validates the authenticity and reliability of our study from a side perspective.

## CONCLUSION

Similar PFS and OS were found between PF and TP regimens with concurrent chemoradiotherapy. Consolidation chemotherapy did not show any significant OS benefits.

## AUTHOR CONTRIBUTIONS

The corresponding authors (Qingshan Zhu and Yuan Tian) designed the study. Chi Zhang, Zhuoqi Li, Tingwei Ma, Nengchao Wan, Weipeng Liu, Zhijie He, Jing Shen, Tao Wei, Shijie Zhao, and Lianjie Feng were responsible for data collection and some formal analysis. The final version of the submission was confirmed by the corresponding author (Qiangshan Zhu) and approved by all participating authors.

## FUNDING INFORMATION

The study was funded by Clinical Research Special Support Fund of Wu Jieping Medical Foundation (320.6570.2023‐16‐12; Yuan Tian), Clinical Research Fund of Shandong Medical Association Qilu Special Project (YXH2022ZX02016; Yuan Tian), Henan Province Medical Science and Technology Research Plan (2 018 021 020; Qingshan Zhu), and Science and Technology Research Plan of Anyang City, Henan Province (2022C01SF003; Qingshan Zhu).

## CONFLICT OF INTEREST STATEMENT

All authors confirmed that no conflict of interest was found.

## Supporting information


**SUPPORTING INFORMATION FIGURE S1.** Short‐term efficacy evaluation results of the two groups. Blue represents Group A, green represents Group B; The horizontal axis represents different therapeutic groups; The vertical axis represents the percentage of occurrence.

**SUPPORTING INFORMATION FIGURE S2.** Imaging findings of patients with esophageal fistula. A: Chest X‐ray of the patient with esophageal fistula. B: Chest X‐ray of the patient after esophageal fistula healing. C: CT image of the patient with esophageal fistula. D: CT image of the patient with esophageal fistula healing after implantation of a nasal feeding tube.

## Data Availability

We promised that all data and information related to the study could be provided; If necessary, you can contact the corresponding author by email.

## References

[1] Bray F , Ferlay J , Soerjomataram I , Siegel RL , Torre LA , Jemal A . Global cancer statistics 2018: GLOBOCAN estimates of incidence and mortality worldwide for 36 cancers in 185 countries. CA Cancer J Clin. 2018;68(6):394–424. 10.3322/caac.21492 30207593

[2] Chen W , Zheng R , Baade PD , Zhang S , Zeng H , Bray F , et al. Cancer statistics in China, 2015. CA Cancer J Clin. 2016;66(2):115–132. 10.3322/caac.21338 26808342

[3] Chen W , Zheng R , Zhang S , Zeng H , Xia C , Zuo T , et al. Cancer incidence and mortality in China, 2013. Cancer Lett. 2017;401:63–71. 10.1016/j.canlet.2017.04.024 28476483

[4] Li F , Li H , Su X , Liang H , Wei L , Shi D , et al. Trends in incidence and mortality of esophageal cancer in China 1990‐2019: a joinpoint and age‐period‐cohort analysis. Front Oncol. 2022;12:887011. 10.3389/fonc.2022.887011 36046041 PMC9420985

[5] Hou H , Meng Z , Zhao X , Ding G , Sun M , Wang W , et al. Survival of esophageal cancer in China: a pooled analysis on hospital‐based studies from 2000 to 2018. Front Oncol. 2019;9:548. 10.3389/fonc.2019.00548 31316913 PMC6610307

[6] Lin Y , Totsuka Y , He Y , Kikuchi S , Qiao Y , Ueda J , et al. Epidemiology of esophageal cancer in Japan and China. J Epidemiol. 2013;23(4):233–242. 10.2188/jea.je20120162 23629646 PMC3709543

[7] Lin Y , Totsuka Y , Shan B , Wang C , Wei W , Qiao Y , et al. Esophageal cancer in high‐risk areas of China: research progress and challenges. Ann Epidemiol. 2017;27(3):215–221. 10.1016/j.annepidem.2016.11.004 28007352

[8] Siegel RL , Miller KD , Jemal A . Cancer statistics, 2018. CA Cancer J Clin. 2018;68(1):7–30. 10.3322/caac.21442 29313949

[9] Ter Veer E , Haj Mohammad N , van Valkenhoef G , Ngai LL , Mali RMA , Anderegg MC , et al. The efficacy and safety of first‐line chemotherapy in advanced esophagogastric cancer: a network meta‐analysis. J Natl Cancer Inst. 2016;108(10). 10.1093/jnci/djw166 27576566

[10] Ando N , Kato H , Igaki H , Shinoda M , Ozawa S , Shimizu H , et al. A randomized trial comparing postoperative adjuvant chemotherapy with cisplatin and 5‐fluorouracil versus preoperative chemotherapy for localized advanced squamous cell carcinoma of the thoracic esophagus (JCOG9907). Ann Surg Oncol. 2012;19(1):68–74. 10.1245/s10434-011-2049-9 21879261

[11] Sun X , Han S , Gu F , Lin G , Wang Z , Wang Y , et al. A retrospective comparison of Taxane and fluorouracil‐based chemoradiotherapy in patients with inoperable esophageal squamous cell carcinoma. J Cancer. 2016;7(9):1066–1073. 10.7150/jca.13547 27326249 PMC4911873

[12] Hu G , Wang Z , Wang Y , Zhang Q , Tang N , Guo J , et al. Comparison of cisplatinum/paclitaxel with cisplatinum/5‐fluorouracil as first‐line therapy for nonsurgical locally advanced esophageal squamous cell carcinoma patients. Drug des Devel Ther. 2016;10:2129–2136. 10.2147/DDDT.S105441 PMC4936807 27445460

[13] Eyck BM , van Lanschot JJB , Hulshof MCCM , van der Wilk BJ , Shapiro J , van Hagen P , et al. CROSS Study Group. Ten‐Year Outcome of Neoadjuvant Chemoradiotherapy Plus Surgery for Esophageal Cancer: The Randomized Controlled CROSS Trial. J Clin Oncol. 2021;39(18):1995–2004. 10.1200/JCO.20.03614 33891478

[14] He L , Allen PK , Potter A , Wang J , Chang JY , Gomez DR , et al. Re‐evaluating the optimal radiation dose for definitive chemoradiotherapy for esophageal squamous cell carcinoma. J Thorac Oncol. 2014;9(9):1398–1405. 10.1097/JTO.0000000000000267 25122435

[15] Siegel RL , Miller KD , Wagle NS , Jemal A . Cancer statistics, 2023. CA Cancer J Clin. 2023;73(1):17–48. 10.3322/caac.21763 36633525

[16] Cooper JS , Guo MD , Herskovic A , Macdonald JS , Martenson JA Jr , Al‐Sarraf M , et al. Chemoradiotherapy of locally advanced esophageal cancer: long‐term follow‐up of a prospective randomized trial (RTOG 85‐01). Radiation Therapy Oncology Group. JAMA. 1999;281(17):1623–1627. 10.1001/jama.281.17.1623 10235156

[17] Yang H , Liu H , Chen Y , Zhu C , Fang W , Yu Z , et al. Long‐term efficacy of neoadjuvant chemoradiotherapy plus surgery for the treatment of locally advanced esophageal squamous cell carcinoma: the NEOCRTEC5010 randomized clinical trial. JAMA Surg. 2021;156(8):721–729. 10.1001/jamasurg.2021.2373 34160577 PMC8223138

[18] Wang T , Yu J , Liu M , Chen Y , Zhu C , Lu L , et al. The benefit of taxane‐based therapies over fluoropyrimidine plus platinum (FP) in the treatment of esophageal cancer: a meta‐analysis of clinical studies. Drug des Devel Ther. 2019;13:539–553. 10.2147/DDDT.S189514 PMC6368118 30787595

[19] Wong IYH , Lam KO , Zhang RQ , Chan WWL , Wong CLY , Chan FSY , et al. Neoadjuvant chemoradiotherapy using cisplatin and 5‐fluorouracil (PF) versus carboplatin and paclitaxel (CROSS regimen) for esophageal squamous cell carcinoma (ESCC): a propensity score‐matched study. Ann Surg. 2020;272(5):779–785. 10.1097/SLA.0000000000004329 32833766

[20] Yang H , Liu H , Chen Y , Zhu C , Fang W , Yu Z , et al. Neoadjuvant chemoradiotherapy followed by surgery versus surgery alone for locally advanced squamous cell carcinoma of the esophagus (NEOCRTEC5010): a phase III multicenter, randomized, open‐label clinical trial. J Clin Oncol. 2018;36(27):2796–2803. 10.1200/JCO.2018.79.1483 30089078 PMC6145832

[21] Shapiro J , van Lanschot JJB , Hulshof MCCM , van Hagen P , van Berge Henegouwen MI , Wijnhoven BPL , et al. CROSS study group. Neoadjuvant chemoradiotherapy plus surgery versus surgery alone for oesophageal or junctional cancer (CROSS): long‐term results of a randomised controlled trial. Lancet Oncol. 2015;16(9):1090–1098. 10.1016/S1470-2045(15)00040-6 26254683

[22] Tang H , Wang H , Fang Y , Zhu JY , Yin J , Shen YX , et al. Neoadjuvant chemoradiotherapy versus neoadjuvant chemotherapy followed by minimally invasive esophagectomy for locally advanced esophageal squamous cell carcinoma: a prospective multicenter randomized clinical trial. Ann Oncol. 2023;34(2):163–172. 10.1016/j.annonc.2022.10.508 36400384

[23] Zhang Y , Li Z , Zhang W , Chen W , Song Y . Risk factors for esophageal fistula in patients with locally advanced esophageal carcinoma receiving chemoradiotherapy. Onco Targets Ther. 2018;11:2311–2317. 10.2147/OTT.S161803 29731639 PMC5923220

[24] Zhu C , Wang S , You Y , Nie K , Ji Y . Risk factors for esophageal fistula in esophageal cancer patients treated with radiotherapy: a systematic review and meta‐analysis. Oncol Res Treat. 2020;43(1–2):34–41. 10.1159/000503754 31639800

[25] Kawakami T , Tsushima T , Omae K , Ogawa H , Shirasu H , Kito Y , et al. Risk factors for esophageal fistula in thoracic esophageal squamous cell carcinoma invading adjacent organs treated with definitive chemoradiotherapy: a monocentric case‐control study. BMC Cancer. 2018;18(1):573. 10.1186/s12885-018-4486-3 29776344 PMC5960135

[26] Coleman RL , Fleming GF , Brady MF , Swisher EM , Steffensen KD , Friedlander M , et al. Veliparib with First‐Line Chemotherapy and as Maintenance Therapy in Ovarian Cancer. N Engl J Med. 2019;381(25):2403–2415. 10.1056/NEJMoa1909707 31562800 PMC6941439

[27] Gomez DR , Blumenschein GR Jr , Lee JJ , Hernandez M , Ye R , Camidge DR , et al. Local consolidative therapy versus maintenance therapy or observation for patients with oligometastatic non‐small‐cell lung cancer without progression after first‐line systemic therapy: a multicentre, randomised, controlled, phase 2 study. Lancet Oncol. 2016;17(12):1672–1682. 10.1016/S1470-2045(16)30532-0 27789196 PMC5143183

[28] Kelly PN . The cancer immunotherapy revolution. Science. 2018;359(6382):1344–1345. 10.1126/science.359.6382.1344 29567702

[29] Wang H , Xu Y , Zuo F , Liu J , Yang J . Immune‐based combination therapy for esophageal cancer. Front Immunol. 2022;13:1020290. 10.3389/fimmu.2022.1020290 36591219 PMC9797857

[30] Shang X , Zhang W , Zhao G , Liang F , Zhang C , Yue J , et al. Pembrolizumab combined with neoadjuvant chemotherapy versus neoadjuvant chemoradiotherapy followed by surgery for locally advanced Oesophageal squamous cell carcinoma: protocol for a multicentre, prospective, randomized‐controlled, phase III clinical study (Keystone‐002). Front Oncol. 2022;12:831345. 10.3389/fonc.2022.831345 35433421 PMC9008846

