# Supplementary figures and images for: The significance of consolidation chemotherapy after concurrent chemoradiotherapy in esophageal squamous cell carcinoma: a randomized controlled phase III clinical trial
Source: Thorac Cancer. 2024 Aug 28;15(28):2038–48. doi: 10.1111/1759-7714.15424 (PMC11444924; doi:10.1111/1759-7714.15424)

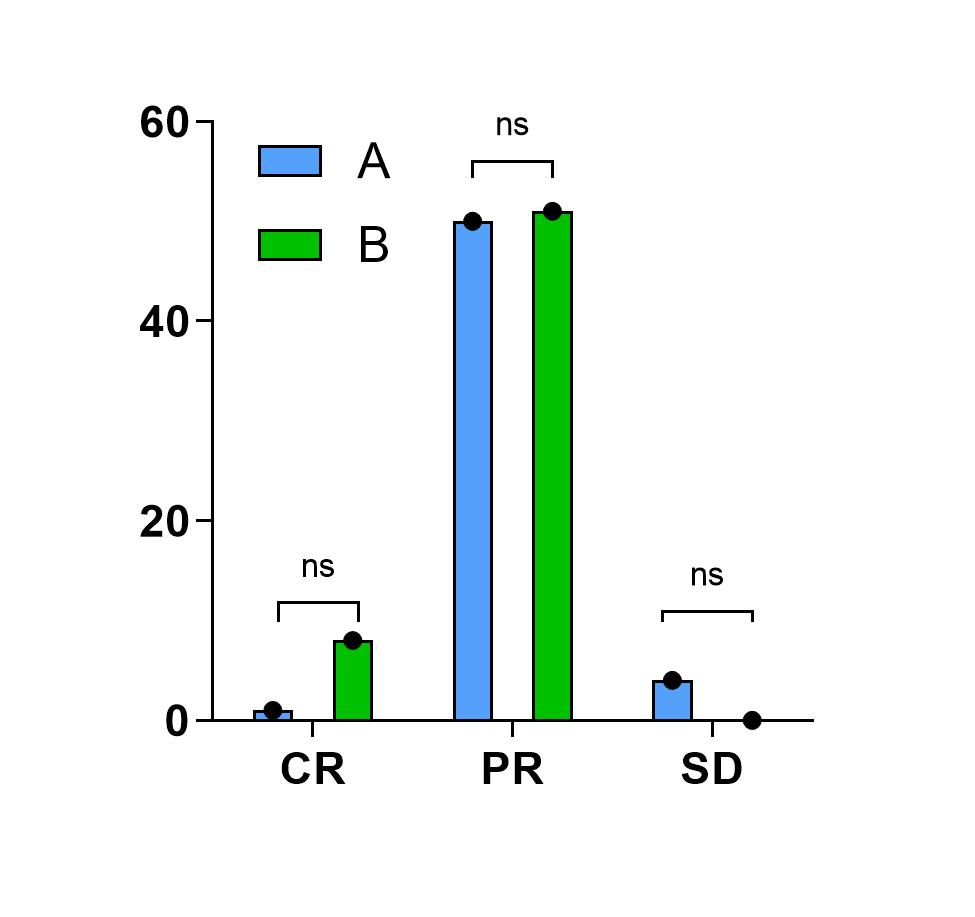

Supplement: Supplementary file 1 — SUPPORTING INFORMATION FIGURE S1. Short‐term efficacy evaluation results of the two groups. Blue represents Group A, green represents Group B; The horizontal axis represents different therapeutic groups; The vertical axis represents the percentage of occurrence. [file TCA-15-2038-s002.tif]

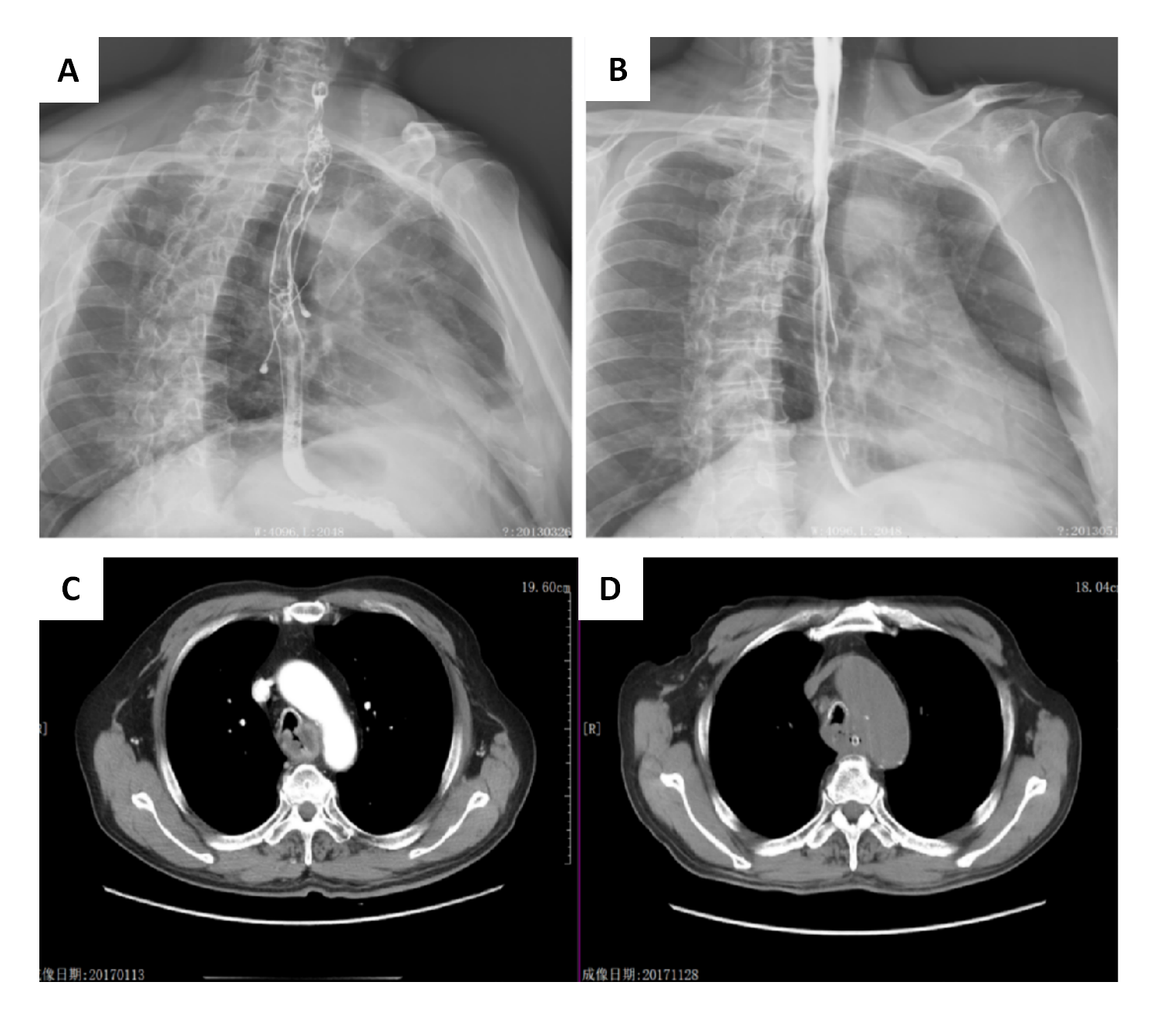

Supplement: Supplementary file 2 — SUPPORTING INFORMATION FIGURE S2. Imaging findings of patients with esophageal fistula. A: Chest X‐ray of the patient with esophageal fistula. B: Chest X‐ray of the patient after esophageal fistula healing. C: CT image of the patient with esophageal fistula. D: CT image of the patient with esophageal fistula healing after implantation of a nasal feeding tube. [file TCA-15-2038-s001.tif]
